# Changes in human gut microbiota composition are linked to the energy metabolic switch during 10 d of Buchinger fasting

**DOI:** 10.1017/jns.2019.33

**Published:** 2019-11-12

**Authors:** Robin Mesnage, Franziska Grundler, Andreas Schwiertz, Yvon Le Maho, Françoise Wilhelmi de Toledo

**Affiliations:** 1 Gene Expression and Therapy Group, King's College London, Faculty of Life Sciences & Medicine, Department of Medical and Molecular Genetics, 8th Floor, Tower Wing, Guy's Hospital, Great Maze Pond, London SE1 9RT, UK; 2 Buchinger Wilhelmi Clinic, Wilhelm-Beck-Straße 27, 88662 Überlingen, Germany; 3 Charité-Universitätsmedizin Berlin, Corporate Member of Freie Universität Berlin, Humboldt-Universität zu Berlin, and Berlin Institut of Health, Berlin, Germany; 4 Institute of Microecology, Auf den Lüppen 8, 35745 Herborn, Germany; 5 Université de Strasbourg, CNRS UMR 7178, Institut Pluridisciplinaire Hubert Curien, 23 rue du Loess, 67200 Strasbourg, France; 6 Département de Biologie Polaire, Centre Scientifique de Monaco, 8 Quai Antoine 1^er^, 98000 Monaco, Monaco

**Keywords:** Periodic fasting, Buchinger fasting, Intestinal permeability, Inflammation, Well-being, BCAA, branched-chain amino acid, BWC, Buchinger Wilhelmi Clinic, EDN, eosinophil-derived neurotoxin, LBP, lipopolysaccharide-binding protein, LPS, lipopolysaccharide, sIgA, secretory IgA

## Abstract

Fasting is increasingly popular to manage metabolic and inflammatory diseases. Despite the role that the human gut microbiota plays in health and diseases, little is known about its composition and functional capacity during prolonged fasting when the external nutrient supply is reduced or suppressed. We analysed the effects of a 10-d periodic fasting on the faecal microbiota of fifteen healthy men. Participants fasted according to the peer-reviewed Buchinger fasting guidelines, which involve a daily energy intake of about 1046 kJ (250 kcal) and an enema every 2 d. Serum biochemistry confirmed the metabolic switch from carbohydrates to fatty acids and ketones. Emotional and physical well-being were enhanced. Faecal 16S rRNA gene amplicon sequencing showed that fasting caused a decrease in the abundance of bacteria known to degrade dietary polysaccharides such as Lachnospiraceae and Ruminococcaceae. There was a concomitant increase in Bacteroidetes and Proteobacteria (*Escherichia coli* and *Bilophila wadsworthia*), known to use host-derived energy substrates. Changes in taxa abundance were associated with serum glucose and faecal branched-chain amino acids (BCAA), suggesting that fasting-induced changes in the gut microbiota are associated with host energy metabolism. These effects were reversed after 3 months. SCFA levels were unchanged at the end of the fasting. We also monitored intestinal permeability and inflammatory status. IL-6, IL-10, interferon γ and TNFα levels increased when food was reintroduced, suggesting a reactivation of the postprandial immune response. We suggest that changes in the gut microbiota are part of the physiological adaptations to a 10-d periodic fasting, potentially influencing its beneficial health effects.

Alternation of food abundance and food scarcity (feast and fast) is part of human and animal physiology. Fasting happens on a daily basis, usually during night hours, but also during short or longer periods of time, e.g. in humans without access to technologies of food conservation^(1)^ or for religious reasons^(2)^. Humans also voluntarily fast because it has been documented to safely enhance well-being and to have therapeutic benefits^(3,4)^. Most scientific investigations in the last decade were, however, focused on intermittent fasting, a recurrence of 16 to 48 h energy restriction alternating with food intake^(3)^. By contrast, very few studies have examined human physiology during a periodic fast lasting from 2 to 21 d or more, such as in the empirically based Buchinger Wilhelmi fasting programme, described in peer-reviewed therapeutic fasting guidelines^(5)^.

When animals or humans switch from eating to periodic fasting, the source of energy for their cells switches from food molecules absorbed through the gastrointestinal tract to the utilisation of energy substrates mobilised out of several body tissues, primarily adipose tissue but also body protein^(6)^. In other words, there is a metabolic switch in energy utilisation from glucose to fatty acids and ketones. This increase in the rate of lipolysis and ketogenesis is reflected by a decrease in blood glucose, insulin, insulin-like growth factor-1, concomitant to an increase in glucagon, growth hormone, NEFA, ketone bodies and insulin-like growth factor binding protein-1 levels^(4,7–9)^.

When the digestive tract is put temporarily at rest during periodic fasting, it remodels its structure leading to a reversible atrophy as shown for rat intestinal villi^(10)^. In addition, changes in gut motility, total intestinal mass^(11)^ and microbiota composition have been described in the gut of fasting animals^(12)^. The reduction of the external nutrient supply to the gut microbiota, and the associated remodelling of the gastrointestinal tissues of the host, have been qualified as a ‘microscopic energy crisis’ coupled to a ‘housing crisis’ for the gut microbiota^(13)^. The gut microbiota relies almost entirely on host diet composition as well as on host food processing capacity to obtain metabolic substrates, and to cover its energy requirements of 150–450 kcal/d (628–1883 kJ/d)^(14)^. It seems therefore inevitable that periods of fasting where no external food enters the digestive tract have repercussions on the microbiota composition.

Changes in gut microbiota caused by fasting have been studied in hibernating animals like hamsters^(15)^, squirrels^(16)^ and brown bears^(17)^. Another study described shared responses of the microbiota across five vertebrates of different classes (tilapia, toads, geckos, quail and mice) during prolonged fasting^(12)^. The most common response was a decrease in the abundance of microbial species using plant glycans as a source of energy, while species using host glycans as a source of energy had their abundance increased^(18)^. Despite the growing interest in several patterns of fasting and particularly periodic fasting, its effects on the human microbial ecosystems remain essentially unknown^(19)^.

Since dietary changes have a large impact on the gut microbiota, it is likely that fasting can also trigger important gut microbiota changes, which may in turn influence host health and immunity^(20,21)^. This has been demonstrated in laboratory animals, already. In mice, intermittent fasting prevents diabetic retinopathy by restructuring the gut microbiota^(22)^. Other publications link therapeutic effects of fasting to changes of gut microbiota in patients with rheumatoid arthritis^(23,24)^. Only one study performed in human subjects showed that after 1 week of fasting, followed by 6 weeks of refeeding and probiotic supplementation, an increase in abundance of lactobacilli, Enterobacteriaceae and *Akkermansia* could be observed within the gut microbiota^(19)^. However, the gut microbiota composition was not measured at the end of the fasting period.

To our knowledge no study so far has been performed to investigate the effects of periodic fasting on the gut microbiota in humans using high-throughput sequencing^(25)^. In order to fill this important gap, we studied the composition of the gut microbiota and a large range of health biomarkers in a cohort of fifteen healthy men before, on the last day of fasting, during the refeeding-period and 3 months after a 10-d periodic Buchinger fasting.

## Materials and methods

### Study design

This study was conducted according to the guidelines laid down in the Declaration of Helsinki and all procedures involving human subjects were approved by the Baden-Württemberg medical council (application no. F-2016-090; 27 September 2016). Written informed consent was obtained from all subjects. The study was registered at the German Clinical Trials Register (DRKS-ID: DRKS00011165, trial registry name: Effects of the Buchinger Wilhelmi fasting programme on energy metabolism and muscle function in humans (https://www.drks.de/drks_web/setLocale_DE.do) 24 October 2016). It was conducted at the Buchinger Wilhelmi Clinic (BWC) in Überlingen, Germany, between 20 November 2016 and 10 December 2016. All participants were in general good health. Four time points were specified for each individual. The baseline examination was conducted 1 d before fasting (time point 1). The second examination was done at the end of the 10-d fasting period (time point 2). The third examination was conducted on the fourth day of the progressive refeeding period after fasting (time point 3). The last examination took place 3 months after the fasting (time point 4). For this follow-up, the subjects returned for 1 d to the BWC between 1 March 2017 and 5 March 2017.

### Participants

**Fig. 1.**
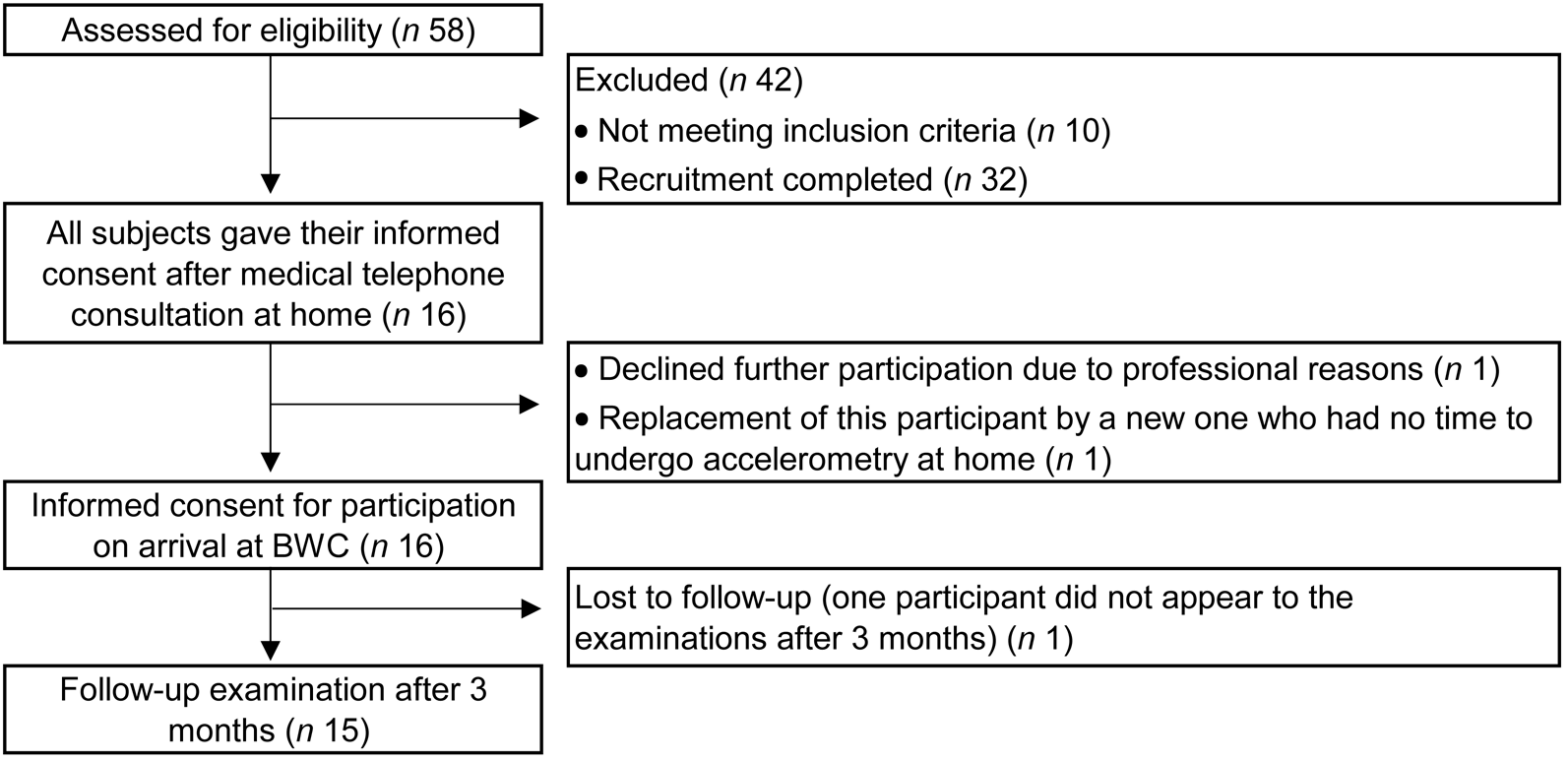
Flow chart of the recruitment procedure. BWC, Buchinger Wilhelmi Clinic.

Participants were recruited in August 2016 among organisations involved in the practice of fasting, which is very popular in Germany. Out of a total of fifty-eight men, we recruited sixteen men according to age, physical and psychological health criteria. Included were men aged between 18 and 70 years with a BMI between 20 and 32 kg/m^2^ (26·5 (sd 3·0) kg/m^2^). One participant was excluded retrospectively due to incomplete collection of stool samples. Thus, the data analysis included fifteen men (Fig. 1). The age of the participants was 44·6 (sd 13·5) years. BMI was 26·5 (sd 3·0) kg/m^2^. Exclusion criteria were predefined according to a list and included cachexia, anorexia nervosa, advanced kidney, liver or cerebrovascular insufficiency^(5)^. Smoking and the intake of antibiotics within the last 8 weeks, as well as the intake of probiotics within the last 4 weeks, led to exclusion.

### Fasting intervention

All subjects fasted according to the fasting programme of the BWC which is documented in the guidelines of the fasting therapy^(5)^. They stayed under daily supervision of nurses and physicians specialised in fasting therapy. On the day of admission in the BWC the participants received a standardised low-carbohydrate vegetarian dinner. On the next day before the beginning of the fast, the participants were given a 600 kcal (2510 kJ) vegetarian diet consisting of rice and vegetables divided in three meals. During fasting all subjects were asked to drink 2–3 litres of water or non-energy herbal teas on a daily basis. Additionally, all participants received a portion of 20 g honey. Furthermore, an organic freshly squeezed fruit juice (250 ml) was served at noon and a vegetable soup (250 ml) in the evening. On average, the total daily nutrient composition is described in Table 1. With the beginning of fasting the subjects entered a standardised programme of physical exercise alternating with rest. The exercise programme consisted of outdoor walks and gymnastic groups. The whole programme was supervised by certified trainers. To initiate the 10-d fasting period, the intestinal tract was emptied through the intake of a laxative (20–40 g NaSO_4_ in 500 ml water according to body weight). During the fasting period an enema (1 litre water at 37°C) was applied by a certified nurse every second day. This procedure, whose effects remains to be investigated in a clinical study, is assumed to remove intestinal remnants of the last meals, desquamated mucosal cells from the gastrointestinal walls and basal secretions both occurring during fasting. Based on clinical and empirical observations, it facilitates the transition to the fasting mode, leads quicker to the fasting-specific absence of hunger, and prevents common symptoms observed at the beginning of the fasting like headaches and fatigue. Although the safety of enemas is still debated, their use for more than 60 years at the BWC has never been linked to clinical complications. From the tenth day of fasting on, food was stepwise reintroduced during the following 4 d. The food consisted of an ovo-lacto-vegetarian organic diet with a progressive increase of energy from 3347 to 6694 kJ/d (800 to 1600 kcal/d) (Supplementary Table S3). The third faecal sample was obtained from the first defecation after refeeding, varying between the first and fourth refeeding day.

**Table 1.** Nutrient composition of the diet during fasting

Parameter	Amount
Fat intake (g/d)	0·2
Protein intake (g/d)	1·8
Carbohydrate intake (g/d)	56·2
Fibre intake (g/d)	1·1
Energy intake	234·4
kJ/d	980·7
kcal/d	234·4

### Clinical parameters

All participants underwent a thorough physical examination, their medical history was documented and their height was assessed in the admission consultation by the physician (Seca 285; Seca). Every morning trained nurses recorded body weight while the subjects wore only underclothing (Seca 704; Seca). Blood pressure and heart frequency were measured at the non-dominant arm in a sitting position (upper-arm blood pressure monitor, boso Carat professional; BOSCH + SOHN GmbH u. Co. KG). Waist circumference was determined with a measuring tape mid-way between the lowest rib and the iliac crest (openmindz® GmbH). The participants self-reported their physical and emotional well-being on numeric rating scales from 0 (very bad) to 10 (excellent). Also, 3-d food protocols were documented prospectively by each subject before fasting and 3 months afterwards. The safety of the Buchinger Wilhelmi periodic fasting programme was continuously monitored by a staff of doctors and nurses.

### Biochemical parameters

Blood samples were collected by trained physicians in the morning and drawn into EDTA (S-Monovette^®^ 2·7 ml K3 EDTA), citrate (S-Monovette^®^ 3 ml 9NC, citrate 3·2 % (1:10)), and blood sedimentation tubes (S-Sedivette^®^ 3·5 ml 4NC, ESR/citrate buffer (1:5)), and were gently shaken after filling. Additionally, serum tubes including serum gel with clotting activator (S-Monovette^®^ 9 ml Z-Gel) were used and stored upright for 30 min until coagulation with a subsequent centrifugation step at 3920 ***g*** for 10 min at room temperature. All tubes were manufactured by Sarstedt AG & Co. The routine parameters were sent to MVZ Labor Ravensburg and analysed according to the manufacturer's instructions in a fully automated laboratory. Blood cell count (leucocytes, erythrocytes, Hb, mean cell volume, thrombocytes) was measured using the blood analyser Sysmex XN-9000 (Sysmex Europe GmbH). Glucose, total cholesterol and TAG were analysed with ADVIA 2400 (Siemens Healthcare GmbH). Insulin was measured with Centaur XP (Siemens Healthcare GmbH). Remaining blood samples were immediately frozen at −70°C.

Inflammatory blood parameters were measured using the Human High Sensitivity T Cell Panel kit (HSTCMAG-28SK). Results were obtained by the Bio-Plex 200 Analyser System and data were analysed with Bio-Plex Manager software (Bio-Rad Laboratories). Bacterial lipopolysaccharide (LPS) serum levels were measured by a commercial ELISA kit (Cusabio). Antibodies specific for LPS were pre-coated onto a microplate and 100 µl of standards or sample were incubated for 2 h at room temperature. After incubation, samples were analysed at 450 nm. Values were expressed as pg/ml; intra-assay and inter-assay CV were 8 and 10 %, respectively. Plasma LPS-binding protein (LBP) was measured by a LBP soluble ELISA kit (Hycult Biotechnology) according to the manufacturer's protocols.

The semi-quantitative concentration of ketone bodies was self-measured in the first morning urine using Ketostix (Bayer AG) that react according to the concentration of acetoacetic acid.

### Faecal parameters

Faecal samples were collected four times using sterile containers (MED AUXIL stool collector set; Süsse). The first sample was collected before the start of the fasting and the second following intake of a laxative (Laxoberal Abführ-Tropfen, sodium picosulphate; Sanofi-Aventis Deutschland GmbH) in the evening of the ninth fasting day. The third sample was obtained from the first defecation after refeeding and the last sample in the follow-up phase. Faecal samples were immediately frozen and stored at −70°C. The samples were sent to the Institute of Microecology for analysis.

Faecal calprotectin and zonulin concentrations were measured by an ELISA as described elsewhere^(26)^. Faecal lactoferrin concentrations were determined using the IBD-SCAN^®^ test (TechLabR, Inc.), faecal α−1-antitrypsin concentrations were analysed using the AAT test (Maier Analytic) following the instructions. Lysozyme and β-defensin were measured by an ELISA (Immunodiagnostik). Branched-chain amino acids (BCAA), EDN (eosinophil-derived neurotoxin, eosinophil protein x), sIgA and bile acid concentration were determined with the BCAA, EDN, IDK^®^ sIgA, and IDK^®^ bile acids test kits, respectively (Immunodiagnostik). SCFA were determined using GC as previously described^(27)^.

### 16S rRNA gene amplicon sequencing

In order to determine the composition of the gut microbiota during the fasting intervention, we sequenced PCR-amplified marker 16S ribosomal RNA genes fragments which contain a bacterial taxa-specific region^(28)^. Microbial DNA was extracted from 200 mg of faecal sample using the QIAsymphony^®^ DSP Virus/Pathogen Mini-Kit (Qiagen) according to the manufacturer's instructions on the QIAsymphony^®^ SP (Qiagen). DNA purity and concentration were measured with an Implen NanoPhotometer P-Class 360 (Implen GmbH).

The partial sequences of the hypervariable region of the 16S rRNA gene (V4 and V5) were PCR amplified using the primer 520 forward (5′-AYTGGGYDTAAAGNG-3′) and 907 reverse (5′-CCGTCAATTCMTTTRAGTTT-3′)^(29)^. PCR amplification was performed at least twice for each sequencing set up. The PCR mixture with a final volume of 25 µl consisted of 0·5 µl of each primer (10 µm), 0·6 µl of dNTP-mix (10 mm each), 5 µl 5× KAPA Hifi Puffer including 20 mm-MgCl_2_ (Roche), 0·1 µl KAPA Hifi Polymerase (Roche), 1 µl DNA isolate and filled up with nuclease-free water. PCR reactions were performed in a T100 Thermal Cycler (Bio-Rad Laboratories) using the following programme: 3 min at 95°C for initial denaturation, twenty-five cycles of 30 s at 95°C for denaturation, 30 s at 55°C for annealing, and 45 s at 72°C for elongation, followed by a final elongation step for 5 min at 72°C. Water-template control and *Escherichia coli* DNA as positive control were included for each set of PCR reactions. Success of PCR were verified by agarose gel electrophoresis using Midori Green as DNA-dye (Biozym). Both PCR were pooled and purified with Agencourt AMPure beads (Beckman Coulter) into 50 µl of 10 mm-Tris (tris(hydroxymethyl)aminomethane; pH 8·5).

A second PCR step was then performed to add unique index barcodes with sequencing adaptors to the amplicon targets. The Ion Torrent set up custom-made index sequences were chosen (added in the supplement; Integrated DNA Technologies) as forward primer. The index PCR reaction had a total volume of 50 µl and included 1 µl Ion-index-primer forward and 1 µl 926Rcomb reverse primer (5′-CCTCTCTATGGGCAGTCGGTGAT CCGTCAATTCMTTTRAGTTT-3′) for Ion-Torrent set ups with 1·2 µl of dNTP-Mix (10 mm each), 10 µl 5× KAPA Hifi Puffer including 20 mm-MgCl_2_ (Roche), 0·2 µl KAPA Hifi Polymerase (Roche), 5 µl amplicon DNA and filled up with nuclease-free water. PCR reactions were performed in a T100 Thermal Cycler (Bio-Rad Laboratories) using the same programme as above with eight cycles. Primers that were used were designed for the V4 and V5 variable region of the bacterial 16S-rRNA gene which led to a length of around 364 bp for bacterial identification. With indices and linker sequences, libraries have a mean sequence length of 528 bp. After purification with AMPure beads, quality checks for library sizes and DNA concentration were performed with the Aglient Bioanalyzer using Aglient DNA 1000 chips (Agilent Technologies). To determine the DNA concentration, the Qubit dsDNA HS Assay Kit (Thermo Fisher Scientific) was used.

Libraries were finally pooled in equivalent 100 pm for Ion Torrent. Libraries prepared for Ion Torrent sequencing template-positive Ion PGM template Hi-Q ion sphere particles were produced using the emulsion PCR technique in the Ion One Touch 2 and the Ion PGM Hi-Q OT2 Kit (Life Technologies) following the manufacturer's instructions for PCR, recovery and quality control. The libraries were divided to be run on four ION 318 chip-kit v2 BC chips (Life Technologies) executing 850 sequencing flows on an Ion PGM sequencer (Life Technologies) following the manufacturer's instructions.

A total of six sequencing runs were performed. Each sequencing run was processed separately in order to account for the differences in sequencing quality. The 16S sequencing data included a total of 31 556 258 reads (average of 498 493 reads per samples, range 70 875–2 884 392) available as demultiplexed FASTQ files on the SRA archive in the project PRJNA531091. Data analysis was done with Rosalind, the BRC/King's College London high-performance computing cluster. The DADA2 algorithm was used^(25)^ to correct for sequencing errors and identify amplicon sequence variants (ASV) using R version 3.5.0. We trimmed the first fifteen poor-quality bases at the 5′ side of reads as recommended by the DADA2 manual^(25)^. A total of 20 728 sequence variants was identified. The taxonomy was assigned using the SiLVA ribosomal RNA gene database v132 up to the species level. ASV with fewer than ten counts in less than 20 % of the individuals were discarded. This resulted in a dataset composed of 1673 ASV in sixty-three individuals that was brought forward to a statistical analysis.

### Statistical analysis

#### Justification of sample size

The sample size was calculated based on the data obtained with Ramadan intermittent fasting in a similar population^(30)^. Assuming a test–retest correlation coefficient for the measure of 0·9, a group of fourteen subjects was necessary to detect a post–pre difference of about 7 % with a power of 0·95 and an α for unilateral test of 0·05.

The α diversity, representing the diversity of the total number of species within the samples^(31)^, was measured using Shannon's diversity index, and the difference between groups evaluated with the Kruskal–Wallis rank sum test. We also measured the β diversity, representing the diversity of the total number of species between the samples^(31)^. We used the Bray–Curtis dissimilarity index which was calculated using the R package *Vegan* and recommended for proportion data. A stress function was used to measure the goodness of fit between the ordination and the original data. The 16S rRNA gene amplicon sequencing dataset was further processed using the R package *Phyloseq* in order to collapse the DNA sequences to different taxonomic levels^(32)^. Relative abundance values were transformed using the centred log-ratio (clr) transformation with the R package *compositions*. We then performed a statistical analysis of the differences in microbiota composition between time points for each taxa and of the associations between taxa abundance and health parameters, by fitting linear mixed models with *lmer*, considering repeated sampling of individuals as random effects. *P* values where calculated using the *difflsmeans* function with the R package *lmertest*. The Benjamini & Hochberg correction procedure was applied to control the false discovery rate^(33)^.

## Results

The aim of this investigation was to describe the changes within the gut microbiota caused by a 10-d fast. This was done by measuring the microbiota composition and clinical parameters 1 d before fasting (time point 1), at the end of the 10-d fasting period (time point 2), on the fourth day of the following progressive refeeding period (time point 3), and 3 months after the fasting period (time point 4). Fasting resulted in a significant weight reduction of 5·9 (sd 0·8) kg (*P* = 0·0002). Abdominal circumference, systolic blood pressure and diastolic blood pressure were also significantly reduced after fasting and at the end of the refeeding period (Table 2). Furthermore, fasting was accompanied by an enhancement of well-being (Table 2). Emotional well-being increased at the end of fasting and was significantly enhanced after the refeeding period (*P* = 0·00079). These parameters returned to a baseline level 3 months after the fasting period. Physical well-being also increased and reached significant enhancement after refeeding (*P* = 0·000094), and was maintained 3 months after fasting (*P* = 0·049).

**Table 2.** Summary of the changes in clinical biomarkers and well-being during fasting (Mean values, ranges and standard deviations)

Parameter	Pre-fasting			End of fasting				3 d after				After 3 months			
	Mean	Range	sd	Mean	Range	sd	*P*	Mean	Range	sd	*P*	Mean	Range	sd	*P*
Weight (kg)	85·7	70·5–106·9	1·0	79·8	66·1–99·5	9·6	***	79·7	66·5–100·1	9·5	***	81·9	70·4–99·5	8·3	NS
BMI (kg/m^2^)	26·5	20·4–32·3	3·0	24·7	19·2–30·0	2·7	***	24·7	19·4–30·1	2·7	***	25·5	21·1–31·8	2·6	*
SBP (mmHg)	128·3	96–158	14·6	121·1	104–152	11·3	*	116·1	103–140	9·6	***	127·6	104–142	10·2	NS
DBP (mmHg)	81·6	57–90	9·3	77·7	64–87	6·5	*	77·6	66–100	9·0	*	81·2	72–95	7·2	NS
Waist (cm)	92·7	76–108	9·1	89·0	74–106	9·0	**	89·7	80–104	7·5	**	92·1	80–108	7·1	NS
Emotional well-being, score 0–10	7·3	4–10	1·9	8·3	2–10	2·1	NS	9·1	6–10	1·2	***	7·9	4–10	1·6	NS
Physical well-being, score 0–10	6·8	3–10	2·4	7·8	1–10	2·4	NS	9·0	5–10	1·5	***	8	5–10	1·4	*

SBP, systolic blood pressure; DBP, diastolic blood pressure.

Significantly different at end of fasting in comparison with pre-fasting baseline levels: *P < 0·05, **P < 0·01, ***P < 0·001 (ANOVA).

### Clinical data measurements

**Fig. 2.**
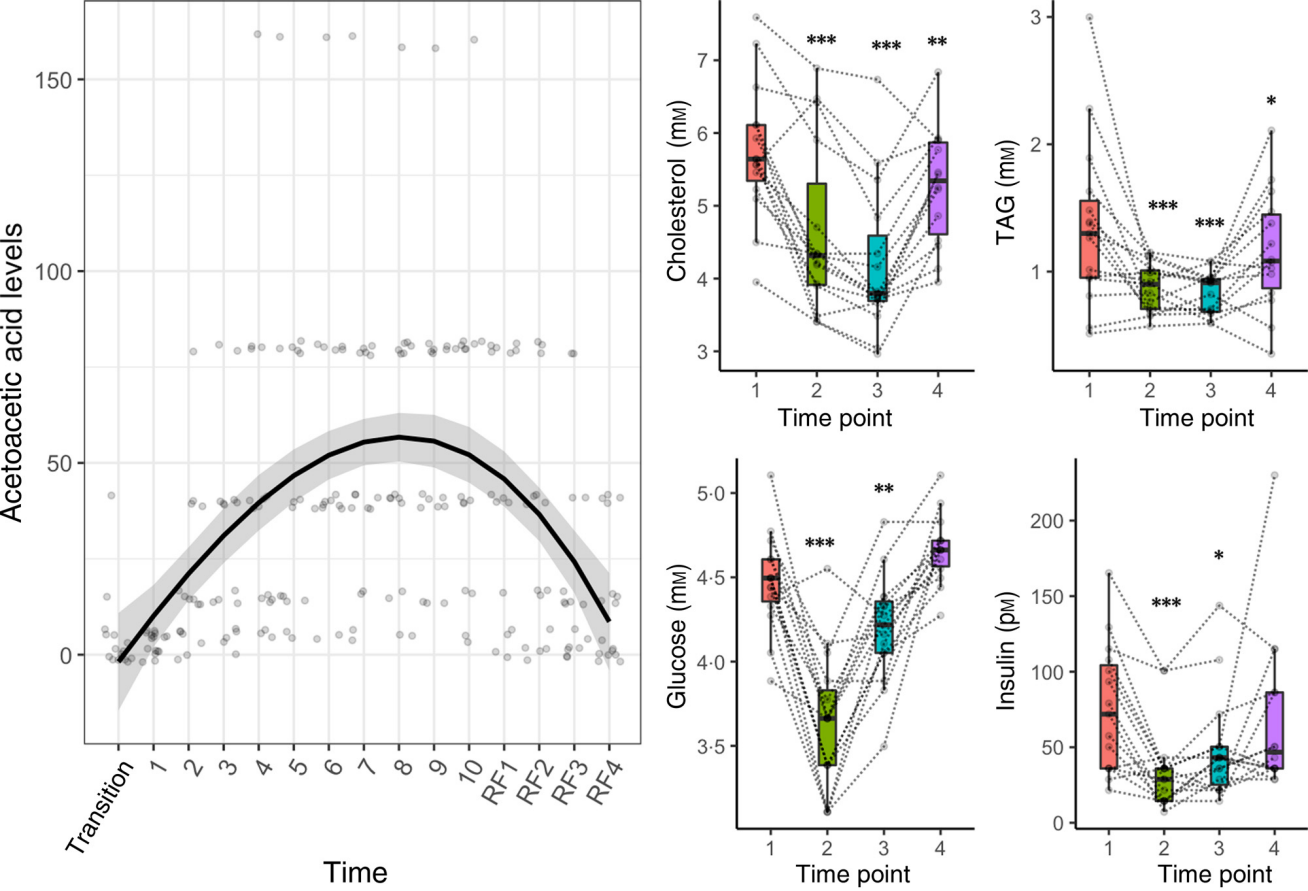
Metabolic switch from carbohydrates to fatty acids and ketones induced by a 10-d fasting. A regression spline was fitted on individual acetoacetic values to show the variations in ketosis during the course of the study. The distribution is summarised by box plots, with the upper and lower hinges extending to the first and third quartiles. Statistical significance was assessed with an ANOVA in comparison with pre-fasting baseline levels (* *P* < 0·05, ** *P* < 0·01, *** *P* < 0·001). Biomarker levels are presented for each individual across the four phases of the intervention (1, baseline examination; 2, at the end of the 10-d fasting period; 3, on the fourth day of the following progressive refeeding (RF); 4, 3 months after the fasting period).

Clinical data measurements confirmed the energy metabolism switch from carbohydrates to fatty acids and ketones (Fig. 2). Glycaemic control improved during fasting: glucose and insulin decreased significantly at the end of fasting and the refeeding period (Fig. 2). Parameters of glucoregulation returned to baseline levels 3 months after the fasting. TAG and total cholesterol were significantly reduced by the fasting period (Fig. 2). Acetoacetic acid in urine increased significantly during fasting (*P* = 1·5 × 10^−6^) and declined during refeeding (*P* = 3·8 × 10^−5^).

### Microbiome analysis

**Fig. 3.**
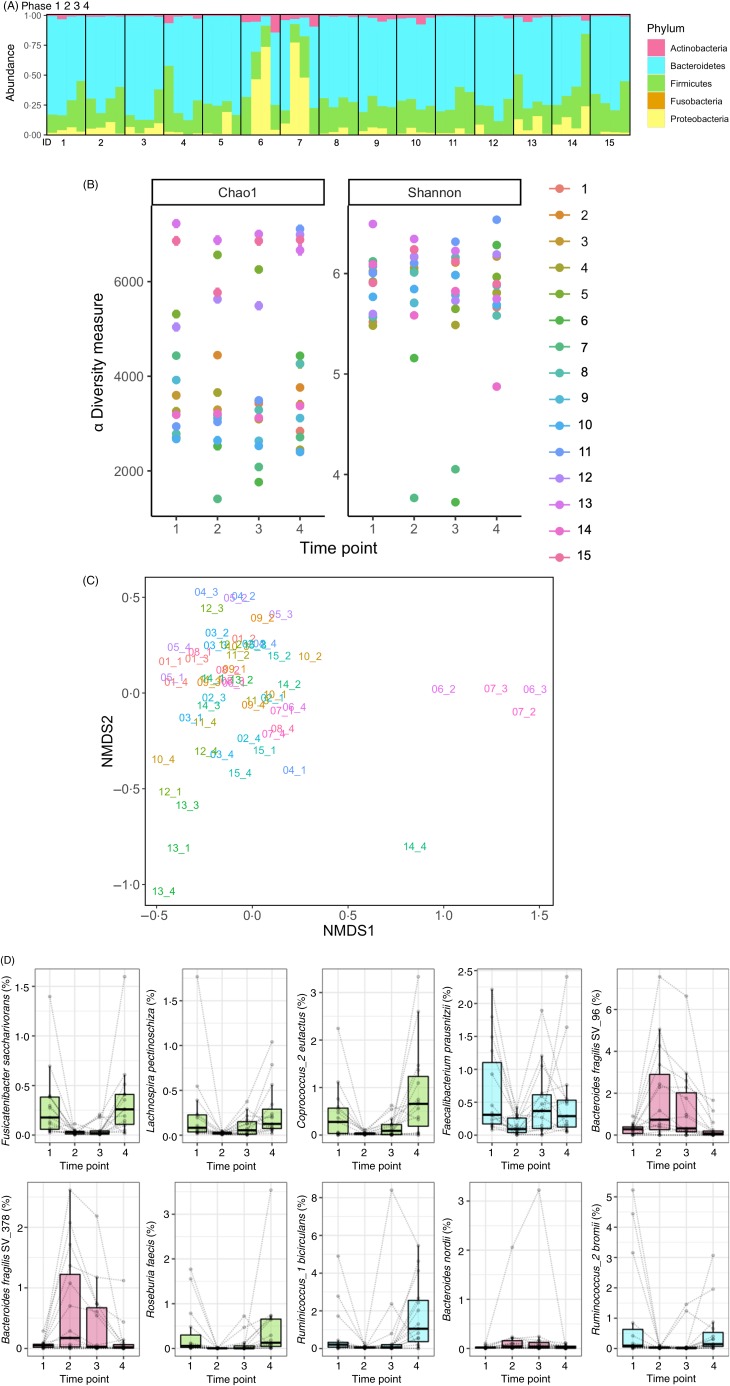
Fasting caused a decrease in the abundance of bacteria from the Lachnospiraceae and Ruminococcaceae families concomitant with an increase in Bacteroidetes. (A) The gut microbiome of fifteen subjects undergoing a 10-d fasting was analysed by 16S rRNA gene amplicon sequencing. The taxonomic profiles are presented for each individual (ID from 1 to 15) across the four phases of the intervention (1, baseline examination; 2, at the end of the 10-d fasting period; 3, on the fourth day of the following progressive refeeding; 4, 3 months after the fasting period). (B) The α diversity was not changed by fasting. (C) The measure of dissimilarities between samples (Bray–Curtis distance) revealed that the samples separate by time point along the *y* axis. (D) The evaluation of changes in species relative abundances across the fasting period revealed that fasting caused a statistically significant decrease in the abundance of bacteria from the Lachnospiraceae family (in green), and from the Ruminococcaceae family (in blue), concomitant with an increase in Bacteroidetes (in pink). The composition of the microbiome returned to a basal level during refeeding. NMDS, non-metric multidimensional scaling.

We identified 213 unique species in the gut microbiota of fifteen individuals. The results from the 16S rRNA gene amplicon sequencing were validated by a taxa-specific quantitative PCR: the Spearman's rank correlations between these two methods for eight taxa were all statistically significant (*P* 0·0016–4·9 × 10^−12^) (Supplementary Fig. S1). The composition of the microbiota was highly individualised (Fig. 3(A)).

There were no differences in α-diversity (Fig. 3(B)). However, the comparison of Bray–Curtis distances revealed that the composition of the microbiota changed during the course of the intervention (Fig. 3(C)). Even if fasting had large effects on the gut microbiome composition, Bray–Curtis distances of the samples for a given individual remained lower than distances across individuals, showing that gut microbiomes remained individualised even after fasting. We then used linear mixed models to identify the bacterial species which were the most affected (Fig. 3(D)). A total of thirty-one sequence variants had their abundances significantly changed by the intervention (Supplementary Table S1). There was an inversion of the Firmicutes:Bacteroidetes ratio (Fig. 3(D)). Bacteroidetes (40·7 %) became the dominant taxa after the fasting period due to a large decrease in the relative abundance of Firmicutes (39·9 %). This included bacteria known to degrade dietary plant polysaccharides such as the Lachnospiraceae family (*Fusicatenibacter saccharivorans*, *Lachnospira pectinoschiza*, *Coprococcus_2 eutactus*, *Pseudobutyrivibrio* spp., *Roseburia faecis*) and from the Ruminococcaceae family (*Faecalibacterium prausnitzii*, *Ruminococcus_1 bicirculans*, *Ruminococcus_2 bromii*). There was a concomitant increase in *Bacteroides* abundance (*Bacteroides nordii*, *Bacteroides fragilis*) and in Proteobacteria abundances (*E. coli*, *Bilophila wadsworthia*). Following food reintroduction (days 10 to 14, from 3347 to 6694 kJ/d (800 to 1600 kcal/d)) the gut microbiota composition reflected a partial recovery (Fig. 3). After 3 months, subjects' gut microbiotas had returned to a basal level in comparison with the baseline established on the day of arrival at the clinic.

### Faecal metabolites

We measured the metabolism of the gut microbiota by measuring the levels of SCFA and BCAA. The concentrations of the main SCFA (acetate, propionate, butyrate) were not changed by the fast. However, a statistically significant decrease in *i*-butyrate (*P* = 0·005) and valerate (*P* = 0·005) levels was observed during the refeeding period. In contrast, the levels of SCFA significantly increased 3 months after the fasting in comparison with pre-intervention levels. This could be linked to the increased abundance of the known SCFA producer *Coprococcus eutactus*. BCAA increased significantly (*P* = 0·00005) during fasting, returned to baseline after refeeding, and declined significantly (*P* = 0·0003) after 3 months (Table 3).

**Table 3.** Serum and faecal biochemistry (Mean values and standard deviations)

Parameter	Pre-fasting		End of fasting			3 d after			After 3 months		
	Mean	sd	Mean	sd	*P*	Mean	sd	*P*	Mean	sd	*P*
Serum biochemistry											
Erythrocytes (10^6^/μl)	5·22	0·55	5·16	0·40	NS	5·03	0·41	NS	5·23	0·48	NS
Hb (mmol/l)	9·48	0·76	9·36	0·45	NS	9·10	0·52	*	9·39	0·59	NS
Haematocrit (%)	44·90	3·27	43·64	2·33	NS	43·75	2·93	NS	44·79	3·00	NS
Leucocytes (10^3^/μl)	6·01	1·38	4·37	1·18	***	3·98	0·93	***	5·5	1·45	NS
MCV (fl)	86·34	4·19	84·75	3·35	NS	87·09	3·06	NS	85·89	4·25	NS
Thrombocytes (10^3^/μl)	234·93	42·28	247·53	41·85	NS	247·13	52·04	NS	258·43	42·36	*
Inflammation parameters in blood											
IFNγ (pg/ml)	78·77	89·43	107·36	70·09	NS	173·95	43·82	***	43·04	21·34	NS
IL-10 (pg/ml)	24·47	14·05	19·67	7·91	NS	34·85	12·06	***	21·88	12·99	NS
IL-6 (pg/ml)	12·57	10·6	17·83	7·89	NS	28·42	7·01	***	13·69	7·03	NS
TNFα (pg/ml)	71·68	79·26	111·03	71·82	NS	165·01	56·9	***	40·41	21·93	NS
LBP (μg/l)	11·82	2·51	9·78	3·80	**	9·58	2·62	**	11·01	1·72	NS
LPS (pg/ml)	15·03	16·29	12·23	13·35	NS	8·93	11·11	NS	8·88	11·65	NS
Inflammation parameters in faeces											
sIgA (ng/ml)	1860·35	1557·2	2812·33	2544·79	NS	3807·01	3013·58	**	1642·06	1148·3	NS
EDN (ng/ml)	404·69	305·63	1176·75	708·56	***	676·6	701·13	NS	437·86	360·56	NS
β-Defensin (ng/ml)	39·17	47·39	61·23	50·28	NS	30·78	27·31	NS	56·46	89·87	NS
Lysozyme (ng/ml)	555·93	304·06	961·33	572·01	*	1183	658·06	**	532·07	236·42	NS
Lactoferrin (μg/g)	1·7	1·7	4·3	12·1	NS	4·8	12·6	NS	1·8	1·8	NS
Calprotectin (μg/g)	29·3	22·2	40·7	71·2	NS	69·3	180·6	NS	26·9	14·2	NS
Bile acid (μmol/100 ml)	287·84	231·04	192·11	131·64	NS	212·13	117·87	NS	291·22	235·97	NS
Gut permeability parameters in faeces											
Zonulin (ng/ml)	74·21	47·69	95·94	107·33	NS	94·13	114·01	NS	107·50	76·05	NS
α-1-Antitrypsin (mg/l)	394	232	520	343	NS	441	389	NS	401	218	NS
Microbial energy metabolism											
Acetate (mm)	63·8	48·3	54·3	33·6	NS	65·9	58·3	NS	100·9	59·4	*
Butyrate (mm)	6·7	6·7	4·8	3·1	NS	4·2	4·8	NS	15·1	13·2	**
*i*-Butyrate (mm)	1·8	1·8	1·4	1·1	NS	0·7	0·7	**	1·7	1·1	NS
Propionate (mm)	9·5	7·4	8·5	4·7	NS	9·6	10·7	NS	17·3	12·8	*
Valerate (mm)	2·7	2·6	2·0	1·6	NS	1·0	1·2	**	2·6	1·9	NS
*i*-Valerate (mm)	1·1	0·8	1·0	0·5	NS	0·6	0·8	NS	1·8	1·2	**
BCAA (μmol/l)	486·58	86·66	575·09	99·24	***	455·51	67·79	NS	403·95	70·35	***

MCV, mean corpuscular volume; IFNγ, interferon γ; LBP, lipopolysaccharide-binding protein; LPS, lipopolysaccharides; sIgA, secretory IgA, EDN, eosinophil-derived neurotoxin; BCAA, branched-chain amino acids.

Significantly different at end of fasting in comparison with pre-fasting baseline levels: *P < 0·05, **P < 0·01, ***P < 0·001 (ANOVA).

### Faecal biochemical markers

The levels in faecal markers of gut permeability (zonulin, α−1-antitrypsin, bile acids) were stable across the different phases of the study (Table 3). Plasma LBP, which reflects the exposure to bacterial LPS, was significantly decreased during fasting and remained reduced during the refeeding period. Faecal markers of intestinal inflammation suggested an inflammatory response after fasting that normalised after 3 months (Table 3).

The levels of IL-6, IL-10, interferon γ and TNFα, which are markers of inflammation, showed a trend towards increase from the beginning to the end of fasting but this increase was not significant. Yet, 4 d after food was reintroduced, there was a significant increase in comparison with baseline levels of all four cytokines (IL-6, IL-10, interferon γ, TNFα) (Table 3). This suggest that an immune reaction associated with inflammation results mainly from food reintroduction.

### Associations between microbiome composition and health markers

**Fig. 4.**
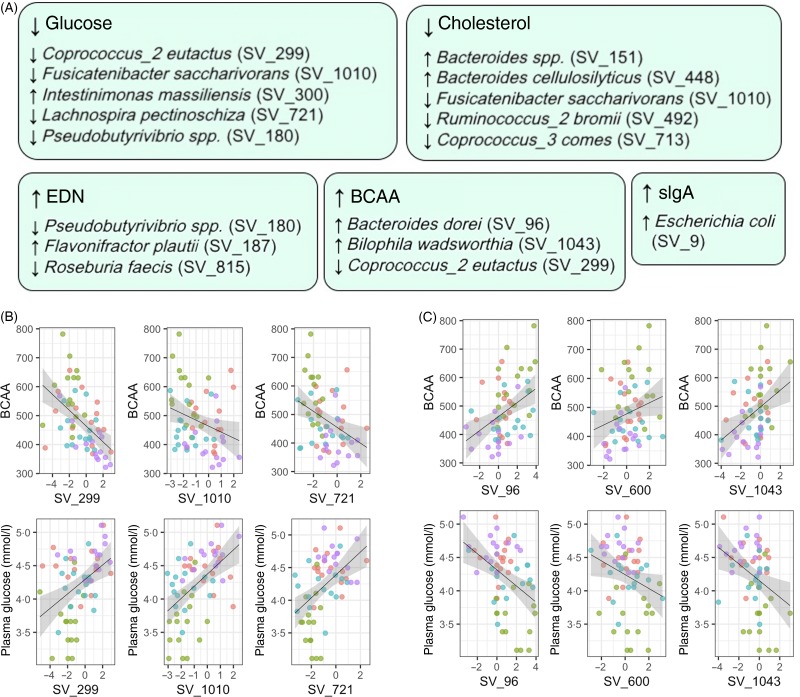
Abundance of bacteria affected by fasting is associated with changes in health biomarkers. The abundance of bacterial species identified by 16S rRNA sequencing was used as a predictor in linear mixed models to understand if they associate with health biomarkers. (A) Statistically significant associations were found between markers of the energy metabolism switch and fasting-affected species. A total of five biochemical parameters are displayed along the bacteria associated with their variations. All arrows indicate statistically significant associations (e.g. a decrease in glucose levels was associated with a decrease in Lachnospiraceae abundance). (B) The abundance in Lachnospiraceae *Coprococcus_2 eutactus* (SV_299), *Fusicatenibacter saccharivorans* (SV_1010) and *Lachnospira pectinoschiza* (SV_721) were consistently positively associated with plasma glucose levels, and negatively associated with branched-chain amino acid (BCAA) levels. By opposition, the Bacteroidaceae *Bacteroides dorei/fragilis* (SV_96) and *Bacteroides thetaiotaomicron* (SV_600), as well as *Bilophila wadsworthia*, presented the opposite trend and were negatively associated with plasma glucose levels, and positively associated with BCAA levels. EDN, eosinophil-derived neurotoxin; sIgA, secretory IgA.

We ultimately evaluated if gut microbiota composition could be associated with variations in markers of health. A total of fifty associations were statistically significant (Benjamini–Hochberg adjusted *P* < 0·05) out of the 4136 associations tested. Although our relatively small sample size only allowed us to draw limited conclusions, the bacterial species which were significantly associated with biochemical parameters were also the bacterial species mainly affected by fasting (Fig. 4). The abundance in Lachnospiraceae (*Coprococcus*_2 *eutactus*, *Fusicatenibacter saccharivorans*, and *Lachnospira pectinoschiza*) was positively associated with plasma glucose levels and negatively associated with BCAA levels. By contrast, Bacteroidetes (*Bacteroides dorei*/*fragilis* and *Bacteroides thetaiotaomicron*), as well as a *Proteobacterium* (*Bilophila wadsworthia*), presented the opposite trend and were negatively associated with plasma glucose levels and positively associated with BCAA levels.

## Discussion

There is a growing interest in biomedical research on fasting and its therapeutic aspects^(7)^, as well as in the gut microbiota and its influence on human health^(34)^. Here we document that periodic fasting in humans has major effects on biochemical markers such as blood lipids, glucoregulation, enhancement of emotional and physical well-being, and the faecal microbiota. We observed a major decrease in the relative abundance of the Firmicutes, Lachnospiraceae and Ruminococcaceae, concomitant to an increase in Bacteroidetes and Proteobacteria. This profile correlates well with known effects of fasting in hibernating animals^(17,18)^ and with daily cyclical fluctuations in the composition of the mouse gut microbiome according to feeding/fasting rhythms^(35)^. When mice are eating, usually during the night, Firmicutes are proliferating and become the dominant phylum while Bacteroidetes rise during fasting (daytime).

The changes in gut microbiome composition and energy metabolism were reversed after 3 months. This reversibility in healthy subjects does not rule out the potential of this fasting protocol to change over the long term the gut microbiota composition of ill subjects. A large number of studies have indicated that unhealthy individuals tend to have a lack of microbial diversity in their gut^(34)^. Since more diverse microbial environments are known to be more resilient^(36)^, we hypothesise that the gut microbiome of unhealthy individuals with a loss of diversity may have a different resilience to the fasting intervention. Prolonged fasting is known to be an efficient therapy in humans to manage metabolic disorders like obesity or high blood pressure^(37)^ and rheumatoid arthritis^(38,39)^. Yet, the present study was performed on healthy subjects and further studies are therefore needed to determine the effects of fasting, and their persistence, on the gut microbiome of patients with specific disorders. Furthermore, fasting can be considered as a metabolic training switching from glucose to fat and ketose and back^(7)^. Healthy subjects gain confidence that fasting is safe and may be used in the case of weight gain, metabolic disorders and other diseases which can be improved by periodic fasting as well as intermittent fasting. When used with a therapeutic goal a special focus is made on the maintenance phase fasting to consolidate the results. Our study lays the foundation for future studies to investigate the impact of fasting on the microbiota of ill persons and their relationship with metabolic and inflammatory mediators.

Furthermore, it is the first to explore the links between the changes in gut microbiome caused by periodic fasting and the changes in inflammation and gut permeability. During periodic Buchinger fasting, almost no nutrients are ingested and consequently absorbed by the small intestine. Therefore, a significant postprandial increase in gut permeability does not occur. This is reflected by the lack of changes in gut permeability markers in our study population in accordance with earlier data^(40)^. However, effects of a periodic fasting on gut permeability may differ between healthy subjects and patients with metabolic disorders. In a previous study, a 4-week energy restriction (800 kcal/d (3347 kJ/d)) in twenty obese women reduced gut permeability^(41)^. Additionally, the gut barrier protects the host from influx of bacterial components such as LPS, components of the outer membrane of Gram-negative bacteria. A biomarker for the translocation of LPS is the LBP^(41)^. In our cohort LBP decreased significantly during fasting, probably reflecting the decreased food intake. Interestingly, although we observed no changes of further inflammation markers during the fasting period, all inflammation markers, except for LPS increases significantly after food reintroduction. This suggests that food intake reactivated the postprandial immune response.

In our cohort, we observed a decrease in blood leucocyte count. This was associated with an initial autophagy triggered by fasting, followed by the activation of bone marrow stem cells^(42)^. The modulation of the immune response by fasting is also shown by the increase in faecal lysozyme during fasting and refeeding, indicating migration of leucocytes into the gut. This is corroborated by the increase in secretory IgA (sIgA), known for their protective action on the mucosal surface^(43)^. An increase in sIgA levels was also demonstrated in a previous study after 17-d Buchinger fasting, and correlated with enhanced immune status^(44)^. Changes in bacterial antigens are described in rheumatoid arthritis^(45)^ concomitant to improvement of the symptoms associated with fasting^(24)^. The association between *E. coli* and sIgA levels detected in our study suggests that the increase in sIgA levels during fasting is due to the increased abundance in bacteria causing an adaptive humoral local response. This is corroborated by recent studies which have shown that commensal Proteobacteria promote a T cell-dependent increase in serum IgA in mice, conferring protection against sepsis^(46)^. In mice, intermittent fasting increases resistance to *Salmonella* infection^(47)^. Since sIgA are known to be the principal eosinophil mediator at mucosal surfaces, causing eosinophil degranulation^(48)^, we could hypothesise that the migration of leucocytes into the gut can also explain the increase in EDN levels during fasting. Altogether, general aspects of the communication between micro-organisms of the digestive tract and the immune system are well described^(49)^.

Although Proteobacteria are generally regarded to have a negative influence on physiological function^(50)^, with *Bilophila wadsworthia* linked to the development of inflammatory bowel disease^(51)^, it is important to note that effects are strain-dependent. For instance, the probiotic *E. coli* Nissle 1917 strain can protect against the invasion by adherent-invasive *E. coli* B2 strains^(52)^. It is thus not possible to definitely attribute beneficial or detrimental health effects to the increase in Proteobacteria abundance observed in our study since the method we used does not allow the determination of gut microbiome composition at the strain level. We recommend new investigations using shotgun metagenomics^(53)^ Moreover, the absence of food intake and thus of digestive processes and absorption during fasting should be taken into consideration when evaluating possible health effects of a bacterial strain. The assessment of physical well-being and gastrointestinal symptomatic outcomes in another study did not indicate a pathological situation^(4)^.

The enhancement of well-being is one of the most contra-intuitive effects of voluntary fasting, whereas involuntary food deprivation is generally experienced as dreadful. The mechanisms underlying this mood enhancement might be similar to those by which physical exercise can induce beneficial responses in the brain, leading to improvements in the processes of learning and memory formation^(54)^. A major contributor is the modulation of brain-derived neurotrophic factor (BDNF) levels by the ketone 3-hydroxybutyrate, as shown on cultured cerebral cortical neurons via an effect on mitochondrial respiration^(55)^. Increasing levels of BDNF levels are also linked to 5-hydroxytryptamine serum levels which are known to increase during fasting, and which thus provide an explanation for positive effects on mood^(53)^. It could also be hypothesised that fasting-induced mood enhancement can be linked to changes in the gut microbiota composition. This hypothesis is corroborated by studies showing that the gut microbiota suppresses *Bdnf* expression in the hypothalamus in mice^(56)^. Moreover, the administration of probiotics is a successful strategy to reduce anxiety and indicates that changes in microbiome profiles have a direct impact on mood^(57)^. As far as well-being during fasting is concerned, additional scientific investigations are needed to understand the role of enemas in preventing symptoms which are often observed at the beginning of the fasting such as headaches and fatigue. Since enemas are known to affect gut microbiome composition^(58)^, it will be important to separate the effects of the enema from the effects of fasting in future studies. Furthermore, an interesting question is whether the observed well-being-enhancing effect of fasting is mainly due to the assumed ability of an enema to remove intestinal remnants, desquamated mucosal cells and fasting basal secretions from the gut or if it results from an effect of the enema on gut microbiota composition.

The gut microbiota may also contribute to weight loss. In our study, the taxonomic group which was most affected by the reduction of nutrient supply was Lachnospiraceae, which is a bacterial family known to have the highest energy-harvesting properties^(59,60)^. Studies demonstrated that a 20 % increase in Firmicutes (i.e. Lachnospiraceae) was associated with an increased energy harvest of about 150 kcal (628 kJ)^(61)^. Since the intrinsic production of energy from the Firmicutes comes in addition to the general energy intake, it can be hypothesised that shutting down Lachnospiraceae-mediated increase of energy uptake had an influence on the weight decrease measured in this study. This is corroborated by the association between Lachnospiraceae abundance and glucose levels. The micro-organisms inhabiting the human gastrointestinal tract have the ability to ferment indigestible complex carbohydrates and produce energy substrates such as SCFA which are then taken up by the host. Up to 5 to 10 % of human energy requirements are covered by SCFA^(62)^. In general, SCFA concentrations remained stable during fasting which is surprising since they are mainly produced by fermentation of dietary fibres^(63)^. Furthermore, an increase in SCFA levels was noticed 3 months after the intervention. Whether this is a consequence of the gut microbiota structural changes due to fasting or a change in dietary habits must be confirmed. Food protocols documented an increase in fibre intake 3 months after fasting (Supplementary Table S2).

The association between Bacteroidetes abundance and faecal BCAA levels in our study suggests that energy requirements in absence of dietary nutrients during fasting could be sustained by the use of host-derived compounds such as desquamated cells. The species which have their levels increased during fasting in our study are also those species known to have the highest proteolytic activity in the gut microbiota^(64)^. The clinical significance of this switch in gut microbiome metabolism is unclear. Experiments in mice showed that the gut microbiome starts feeding on host mucins and degrade the colonic mucus barrier when it is deprived of dietary fibres^(65)^. Little is known about the fate of the mucus barrier during fasting in humans. Starved broilers (also deprived of water) have a thinner mucus adherent layer throughout the small intestine^(66)^. However, this might not reflect physiology during prolonged fasting since the gut is known to undertake substantial structural changes^(11)^. It should also be taken into account that the microbial density is severely reduced during fasting. During hibernation, microbial density decreases by 93·7 % in the caecum of hamsters^(15)^. This cannot be evaluated in our study because the sequencing of 16S rRNA gene amplicons is not quantitative, and because faecal microbiota is not fully reflecting the intestinal microbiota.

In conclusion, 10 d of fasting led to a profound change within the gut microbiota composition and function. The reductions in body weight and waist circumference were associated with an enhancement of well-being and an improvement of energy metabolism. Refeeding induced an immune reaction, as shown by circulating cytokines. This study on healthy subjects lays the foundation for further investigations on the impact of fasting on the microbiota and their relationship with metabolic and inflammatory mediators in diet-related chronic diseases.

## Supplementary material

For supplementary material accompanying this paper visit https://doi.org/10.1017/jns.2019.33. click here to view supplementary material
