# Supplementary material for: Changes in human gut microbiota composition are linked to the energy metabolic switch during 10 d of Buchinger fasting
Source: J Nutr Sci. 2019 Nov 12;8:e36. doi: 10.1017/jns.2019.33 (PMC6861737; doi:10.1017/jns.2019.33)
Supplement: Supplementary file 1 [file S2048679019000338sup.zip › S2048679019000338sup001.doc]

**Supplementary material**

**Supplementary Fig. S1.** The results of the 16S rRNA gene sequencing analysis are validated by qPCR. Spearman's rank correlations between bacterial abundance determined by 16S rRNA gene amplicon sequencing and by taxa-specific qPCR for 8 taxa were all statistically significant. P-values for the statistical significance of Spearman rank correlation coefficients ranged between 0·0016 and 4·9x10^-12^.

**Supplementary Table S1. Summary statistics of amplicon sequence variants having their abundance altered during a long-term ten-day fasting.** P values from linear mixed models were adjusted using the Benjamini & Hochberg correction procedure was applied to control the false discovery rate. The coefficients ß indicate the variations in abundance for the different ASV across the different phases of the study (ß_1_2, changes after ten-days of fasting ; ß_1_3, changes after progressive refeeding ; ß_1_4, changes after 3 months) in comparison to the control baseline levels. Full details are available as a supplementary material.

| **SV_ID** | **Genus** | **Species** | **P** | **Adj_P** | **ß_1_2** | **ß_1_3** | **ß_1_4** |
| --- | --- | --- | --- | --- | --- | --- | --- |
| SV_299 | Phascolarctobacterium | succinatutens | 2·9E-03 | 2·8E-02 | -0·5 | -1·2 | -1·2 |
| SV_1010 | Bacteroides | dorei/fragilis | 5·5E-06 | 3·5E-04 | 1·5 | 0·9 | -0·8 |
| SV_96 | Bacteroides | nordii | 9·7E-05 | 2·1E-03 | 1·5 | 2·1 | 0·5 |
| SV_229 | Bacteroides | fragilis | 1·8E-03 | 2·0E-02 | 1·6 | 0·8 | 0·0 |
| SV_721 | Bacteroides | faecichinchillae/faecis/finegoldii/thetaiotaomicron | 2·6E-03 | 2·8E-02 | 0·8 | 0·4 | -1·0 |
| SV_180 | Bacteroides | fragilis/ovatus | 7·3E-03 | 4·7E-02 | -0·8 | 0·4 | -0·2 |
| SV_713 | Coprobacter | fastidiosus | 3·2E-03 | 2·9E-02 | 0·3 | 1·1 | -0·7 |
| SV_859 | Sutterella | wadsworthensis | 5·5E-03 | 3·7E-02 | -0·6 | 0·3 | -1·1 |
| SV_896 | Granulicatella | adiacens/para-adiacens | 3·8E-03 | 3·0E-02 | 0·8 | -0·2 | -0·9 |
| SV_1043 | Bilophila | wadsworthia | 2·0E-04 | 3·8E-03 | 0·9 | 0·2 | -0·8 |
| SV_815 | Escherichia/Shigella | albertii/boydii/coli […] | 5·2E-04 | 7·7E-03 | 1·7 | 1·4 | -0·3 |
| SV_9 | Coprococcus_2 | eutactus | 1·0E-06 | 1·0E-04 | -1·7 | -0·2 | 1·1 |
| SV_780 | Fusicatenibacter | saccharivorans | 7·1E-07 | 1·0E-04 | -1·9 | -1·7 | 0·1 |
| SV_300 | Lachnospira | pectinoschiza | 1·4E-05 | 5·3E-04 | -1·6 | -0·8 | 0·4 |
| SV_492 | Pseudobutyrivibrio | ruminis/xylanivorans | 1·7E-05 | 5·6E-04 | -2·0 | -1·4 | 0·8 |
| SV_604 | Coprococcus_3 | comes | 3·4E-05 | 9·5E-04 | -0·5 | -1·2 | 0·7 |
| SV_378 | Roseburia | faecis | 4·3E-04 | 7·5E-03 | -2·2 | -0·9 | 0·4 |
| SV_95 | Roseburia | intestinalis | 3·7E-03 | 3·0E-02 | -0·9 | 0·1 | 0·7 |
| SV_1476 | Butyricimonas | paravirosa | 4·8E-04 | 7·7E-03 | 1·2 | 0·9 | -0·4 |
| SV_366 | Romboutsia | ilealis/timonensis | 3·5E-03 | 3·0E-02 | -1·2 | -1·1 | 0·8 |
| SV_1180 | Ruminococcus_1 | bicirculans | 1·0E-05 | 5·0E-04 | -1·5 | -0·1 | 1·0 |
| SV_394 | Intestinimonas | massiliensis | 7·6E-04 | 1·0E-02 | 1·6 | 1·2 | -0·5 |
| SV_536 | Ruminococcus_2 | bromii | 8·4E-04 | 1·1E-02 | -2·1 | -1·7 | 0·1 |
| SV_2432 | Faecalibacterium | prausnitzii | 1·1E-03 | 1·4E-02 | -1·7 | -0·5 | -0·3 |
| SV_558 | Intestinimonas | butyriciproducens | 4·2E-03 | 3·1E-02 | 1·4 | 0·9 | -0·1 |
| SV_2470 | Flavonifractor | plautii | 4·5E-03 | 3·2E-02 | 1·5 | 1·0 | -0·1 |
| SV_187 | Anaerotruncus | colihominis | 8·0E-03 | 5·0E-02 | 1·4 | 1·2 | 0·2 |
| SV_38 | Streptococcus | anginosus/australis/cristatus/infantis […] | 9·7E-05 | 2·1E-03 | 1·9 | 0·1 | 0·3 |
| SV_117 | Streptococcus | anginosus/australis/infantis/mitis/oralis […] | 2·7E-03 | 2·8E-02 | 1·5 | -0·2 | 0·1 |
| SV_287 | Streptococcus | anginosus/mitis/oralis/pneumoniae […] | 4·2E-03 | 3·1E-02 | 0·9 | 0·2 | -0·3 |
| SV_1233 | Dialister | invisus | 5·1E-03 | 3·5E-02 | -0·8 | -0·8 | 0·4 |

**Supplementary Table S2.** Relation of the macronutrients calculated from 3-day food records before and 3 months after fasting

|  |  | **calories (in kcal)** | | | **fibres (in g)** | | | **carbohydrates (in g)** | | | **proteins (in g)** | | | **fat (in g)** | | |
| --- | --- | --- | --- | --- | --- | --- | --- | --- | --- | --- | --- | --- | --- | --- | --- | --- |
| **ID** | **Day** | **pre-fasting** | **after 3 months** | **change** | **pre-fasting** | **after 3 months** | **change** | **pre-fasting** | **after 3 months** | **change** | **pre-fasting** | **after 3 months** | **change** | **pre-fasting** | **after 3 months** | **change** |
| 1 | 1 | 2850 | 3820 |  | 49 | 44 |  | 350 | 385 |  | 82 | 171 |  | 93 | 162 |  |
| 1 | 2 | 3120 | 3390 |  | 46 | 48 |  | 376 | 375 |  | 94 | 165 |  | 123 | 123 |  |
| 1 | 3 | 4310 | 2440 |  | 89 | 37 |  | 582 | 256 |  | 138 | 133 |  | 124 | 87 |  |
| **1** | **mean** | **3427** | **3217** | **-210** | **61** | **43** | **-18** | **436** | **339** | **-97** | **105** | **156** | **52** | **113** | **124** | **11** |
| 2 | 1 | 1800 | 2220 |  | 24 | 17 |  | 196 | 166 |  | 48 | 106 |  | 85 | 111 |  |
| 2 | 2 | 1750 | 1750 |  | 17 | 10 |  | 121 | 121 |  | 76 | 76 |  | 94 | 94 |  |
| 2 | 3 | 2060 | 2100 |  | 24 | 18 |  | 183 | 200 |  | 53 | 105 |  | 113 | 81 |  |
| **2** | **mean** | **1870** | **2023** | **153** | **64** | **45** | **-19** | **167** | **162** | **-4** | **59** | **96** | **37** | **97** | **95** | **-2** |
| 3 | 1 | 4480 | 5130 |  | 47 | 77 |  | 329 | 450 |  | 140 | 157 |  | 277 | 243 |  |
| 3 | 2 | 3730 | 3380 |  | 48 | 61 |  | 422 | 364 |  | 112 | 135 |  | 163 | 123 |  |
| 3 | 3 | 4360 | 3450 |  | 49 | 67 |  | 432 | 344 |  | 108 | 125 |  | 218 | 129 |  |
| **3** | **mean** | **4190** | **3987** | **-203** | **48** | **68** | **20** | **394** | **386** | **-8** | **120** | **139** | **19** | **219** | **165** | **-54** |
| 4 | 1 | 1500 | 611 |  | 7 | 14 |  | 118 | 84 |  | 67 | 22 |  | 82 | 16 |  |
| 4 | 2 | 1460 | 1490 |  | 7 | 16 |  | 128 | 149 |  | 85 | 78 |  | 64 | 58 |  |
| 4 | 3 | 1310 | 709 |  | 15 | 9 |  | 159 | 65 |  | 62 | 57 |  | 41 | 21 |  |
| **4** | **mean** | **1423** | **937** | **-487** | **10** | **13** | **3** | **135** | **99** | **-36** | **71** | **53** | **-19** | **62** | **32** | **-30** |
| 5 | 1 | 2090 | 2540 |  | 27 | 36 |  | 83 | 161 |  | 103 | 103 |  | 140 | 156 |  |
| 5 | 2 | 1810 | 1980 |  | 33 | 21 |  | 96 | 117 |  | 86 | 111 |  | 110 | 111 |  |
| 5 | 3 | 2540 | 4140 |  | 38 | 50 |  | 88 | 147 |  | 131 | 147 |  | 177 | 318 |  |
| **5** | **mean** | **2147** | **2887** | **740** | **33** | **36** | **3** | **89** | **142** | **53** | **107** | **120** | **14** | **142** | **195** | **53** |
| 6 | 1 | 1840 | 2750 |  | 17 | 22 |  | 213 | 346 |  | 71 | 81 |  | 72 | 103 |  |
| 6 | 2 | 2690 | 3640 |  | 13 | 26 |  | 328 | 568 |  | 85 | 119 |  | 107 | 78 |  |
| 6 | 3 | 2340 | 3950 |  | 13 | 34 |  | 198 | 565 |  | 98 | 113 |  | 125 | 116 |  |
| **6** | **mean** | **2290** | **3447** | **1157** | **14** | **27** | **13** | **246** | **493** | **247** | **85** | **104** | **20** | **101** | **99** | **-2** |
| 7 | 1 | 3100 | 1220 |  | 33 | 18 |  | 414 | 122 |  | 103 | 52 |  | 102 | 53 |  |
| 7 | 2 | 1900 | 2240 |  | 19 | 49 |  | 184 | 293 |  | 112 | 92 |  | 73 | 65 |  |
| 7 | 3 | 3130 | 2080 |  | 59 | 33 |  | 378 | 226 |  | 122 | 67 |  | 96 | 83 |  |
| **7** | **mean** | **2710** | **1847** | **-863** | **37** | **33** | **-4** | **325** | **214** | **-112** | **112** | **70** | **-42** | **90** | **67** | **-24** |
| 8 | 1 | 1630 | 2250 |  | 12 | 31 |  | 171 | 299 |  | 40 | 94 |  | 68 | 67 |  |
| 8 | 2 | 5880 | 2540 |  | 43 | 13 |  | 717 | 299 |  | 172 | 52 |  | 219 | 107 |  |
| 8 | 3 | 4210 | 2200 |  | 33 | 7 |  | 437 | 305 |  | 12 | 56 |  | 191 | 65 |  |
| **8** | **mean** | **3907** | **2330** | **-1577** | **29** | **17** | **-12** | **442** | **301** | **-141** | **75** | **67** | **-8** | **159** | **80** | **-80** |
| 9 | 1 | 2330 | 2600 |  | 9 | 20 |  | 143 | 260 |  | 78 | 80 |  | 120 | 94 |  |
| 9 | 2 | 1190 | 2500 |  | 9 | 22 |  | 151 | 259 |  | 30 | 79 |  | 47 | 119 |  |
| 9 | 3 | 2400 | 2010 |  | 12 | 14 |  | 264 | 242 |  | 72 | 94 |  | 66 | 69 |  |
| **9** | **mean** | **1973** | **2370** | **397** | **10** | **19** | **9** | **186** | **254** | **68** | **60** | **84** | **24** | **78** | **94** | **16** |
| 10 | 1 | 4430 | 2540 |  | 27 | 18 |  | 486 | 256 |  | 138 | 128 |  | 138 | 94 |  |
| 10 | 2 | 2190 | 2720 |  | 20 | 22 |  | 281 | 309 |  | 83 | 120 |  | 73 | 103 |  |
| 10 | 3 | 2570 | 2990 |  | 18 | 29 |  | 209 | 324 |  | 102 | 91 |  | 140 | 107 |  |
| **10** | **mean** | **3063** | **2750** | **-313** | **22** | **23** | **1** | **325** | **296** | **-29** | **108** | **113** | **6** | **117** | **101** | **-16** |
| 11 | 1 | 3860 | 2440 |  | 36 | 31 |  | 326 | 229 |  | 205 | 119 |  | 184 | 108 |  |
| 11 | 2 | 3400 | 3650 |  | 45 | 49 |  | 326 | 205 |  | 97 | 202 |  | 125 | 211 |  |
| 11 | 3 | 2480 | 4010 |  | 22 | 56 |  | 4 | 392 |  | 2 | 158 |  | 1 | 186 |  |
| **11** | **mean** | **3247** | **3367** | **120** | **34** | **45** | **11** | **219** | **275** | **57** | **101** | **160** | **58** | **103** | **168** | **65** |
| 12 | 1 | 2380 | 1800 |  | 24 | 41 |  | 270 | 283 |  | 68 | 35 |  | 107 | 30 |  |
| 12 | 2 | 2550 | 3120 |  | 23 | 28 |  | 316 | 463 |  | 82 | 70 |  | 100 | 85 |  |
| 12 | 3 | 3630 | 1840 |  | 22 | 29 |  | 466 | 281 |  | 100 | 49 |  | 142 | 38 |  |
| **12** | **mean** | **2853** | **2253** | **-600** | **23** | **32** | **10** | **351** | **342** | **-8** | **83** | **51** | **-32** | **116** | **51** | **-65** |
| 13 | 1 | 2940 | 4130 |  | 58 | 73 |  | 396 | 503 |  | 93 | 193 |  | 92 | 119 |  |
| 13 | 2 | 3020 | 3430 |  | 65 | 77 |  | 432 | 482 |  | 104 | 117 |  | 56 | 81 |  |
| 13 | 3 | 2820 | 3020 |  | 58 | 53 |  | 384 | 416 |  | 89 | 144 |  | 77 | 59 |  |
| **13** | **mean** | **2927** | **3527** | **600** | **60** | **68** | **8** | **404** | **467** | **63** | **95** | **151** | **56** | **75** | **87** | **11** |
| 14 | 1 | 3160 | 1800 |  | 37 | 14 |  | 270 | 7 |  | 68 | 1 |  | 107 | 1 |  |
| 14 | 2 | 4050 | 3290 |  | 41 | 37 |  | 316 | 463 |  | 82 | 70 |  | 100 | 85 |  |
| 14 | 3 | 2870 | 3530 |  | 32 | 45 |  | 466 | 281 |  | 100 | 49 |  | 142 | 38 |  |
| **14** | **mean** | **3360** | **2873** | **-487** | **37** | **32** | **-5** | **351** | **250** | **-100** | **83** | **40** | **-43** | **116** | **41** | **-75** |
| 15 | 1 | — | 3460 |  | — | 33 |  | — | 319 |  | — | 89 |  | — | 194 |  |
| 15 | 2 | — | 1930 |  | — | 30 |  | — | 206 |  | — | 64 |  | — | 87 |  |
| 15 | 3 | — | 1540 |  | — | 18 |  | — | 155 |  | — | 79 |  | — | 61 |  |
| **15** | **mean** | — | **2310** | — | — | **27** | — | — | **227** | — | — | **77** | — | — | **114** | — |

**Supplementary Table S3.** Diet during the refeeding period and its nutrient composition

|  | **F10** | **RF1** | **RF2** | **RF3** | **RF4** |
| --- | --- | --- | --- | --- | --- |
|  | breaking the fast | refeeding day 1 | refeeding day 2 | refeeding day 3 | refeeding day 4 |
| **Breakfast** | — | 2 prunes, oat porridge with apple | 2 prunes, muesli | 1 piece of fruit, muesli, crispbread with vegetarian spreads | muesli, bread with vegetarian spreads |
| **Lunch** | apple compote | chicory salad, quark and linseed oil cream, mashed potatoes, fresh spinach | leaf salad, quark and linseed oil cream, quinoa soufflé, tomatoes with aubergine caviar with courgette foam | Sauerkraut and leaf salad, potatoes and beetroot with quark and linseed oil cream | Normocaloric individual nutritional program |
| **Intermeal** | 1 raw apple | 1 low-fat yoghurt, 4 nuts | 1 low-fat yoghurt, 4 nuts | 1 low-fat yoghurt, 4 nuts |  |
| **Dinner** | potato and vegetable soup | pumpkin soup, vegetables with rice balls | fennel salad, braised chicory with vegetable jus and bulgur wheat | celery salad with walnut marinade, pumpkin soufflé with spicy dill sauce and vegetables |  |
| **Fat intake (g/day)** | 5 | 22,3 | 32,0 | 47,9 |  |
| **Protein intake (g/day)** | 4 | 31,6 | 28,7 | 57,1 |  |
| **Carbohydrate intake (g/day)** | 50 | 150 | 116 | 149 |  |
| **Fibre intake (g/day)** | 9,3 | 28,4 | 28,9 | 29,8 |  |
| **Energy intake (kcal/day)** | 285 | 999 | 935 | 1340 |  |
